# Metabolic profile of blood serum in experimental arterial hypertension

**DOI:** 10.18699/VJGB-23-64

**Published:** 2023-09

**Authors:** A.A. Seryapina, A.A. Malyavko, Yu.K. Polityko, L.V . Yanshole, Yu.P. Tsentalovich, A.L. Markel

**Affiliations:** Institute of Cytology and Genetics of the Siberian Branch of the Russian Academy of Sciences, Novosibirsk, Russia; Institute of Cytology and Genetics of the Siberian Branch of the Russian Academy of Sciences, Novosibirsk, Russia; Institute of Cytology and Genetics of the Siberian Branch of the Russian Academy of Sciences, Novosibirsk, Russia; International Tomography Center of the Siberian Branch of the Russian Academy of Sciences, Novosibirsk, Russia; International Tomography Center of the Siberian Branch of the Russian Academy of Sciences, Novosibirsk, Russia; Novosibirsk State University, Novosibirsk, Russia

**Keywords:** arterial hypertension, ISIAH rats, metabolic markers, артериальная гипертензия, крысы НИСАГ (ISIAH), метаболомные маркеры

## Abstract

The etiology of essential hypertension is intricate, since it employs simultaneously various body systems related to the regulation of blood pressure in one way or another: the sympathetic nervous system, renin-angiotensin-aldosterone and hypothalamic-pituitary-adrenal systems, renal and endothelial mechanisms. The pathogenesis of hypertension is influenced by a variety of both genetic and environmental factors, which determines the heterogeneity of the disease in human population. Hence, there is a need to perform research on experimental models – inbred animal strains, one of them being ISIAH rat strain, which is designed to simulate inherited stress-induced arterial hypertension as close as possible to primary (or essential) hypertension in humans. To determine specific markers of diseases, various omics technologies are applied, including metabolomics, which makes it possible to evaluate the content of low-molecular compounds – amino acids, lipids, carbohydrates, nucleic acids fragments – in biological samples available for clinical analysis (blood and urine). We analyzed the metabolic profile of the blood serum of male ISIAH rats with a genetic stress-dependent form of arterial hypertension in comparison with the normotensive WAG rats. Using the method of nuclear magnetic resonance spectroscopy (NMR spectroscopy), 56 metabolites in blood serum samples were identified, 18 of which were shown to have significant interstrain differences in serum concentrations. Statistical analysis of the data obtained showed that the hypertensive status of ISIAH rats is characterized by increased concentrations of leucine, isoleucine, valine, myo-inositol, isobutyrate, glutamate, glutamine, ornithine and creatine phosphate, and reduced concentrations of 2-hydroxyisobutyrate, betaine, tyrosine and tryptophan. Such a ratio of the metabolite concentrations is associated with changes in the regulation of glucose metabolism (metabolic markers – leucine, isoleucine, valine, myo-inositol), of nitric oxide synthesis (ornithine) and catecholamine pathway (tyrosine), and with inflammatory processes (metabolic markers – betaine, tryptophan), all of these changes being typical for hypertensive status. Thus, metabolic profiling of the stress-dependent form of arterial hypertension seems to be an important result for a personalized approach to the prevention and treatment of hypertensive disease.

## Abstract (ru)

Этиология гипертонической болезни неочевидна, поскольку одновременно оказываются задействованы различные системы организма, тем или иным образом связанные с регуляцией артериального давления: симпатическая нервная, ренин-ангиотензин-альдостероновая и гипоталамо-гипофизарно-надпочечниковая системы, почечные и эндотелиальные механизмы. На патогенез гипертонической болезни влияет множество как генетических, так и средовых факторов, что обусловливает популяционную гетерогенность заболевания у людей. В связи с этим возникает необходимость в проведении исследований на экспериментальных моделях – инбредных линиях животных. Таковой является линия крыс НИСАГ (ISIAH), воспроизводящая наследственную индуцированную стрессом артериальную гипертензию, максимально приближенную к артериальной гипертонии у людей. Для определения специфических маркеров заболеваний используются «омиксные» технологии, в том числе метаболомные, которые дают представление о профиле концентраций низкомолекулярных соединений – аминокислот, липидов, углеводов, фрагментов нуклеиновых кислот – в биологических образцах, доступных для клинического анализа (кровь и моча). В настоящей работе проведен анализ метаболомного профиля сыворотки крови самцов крыс линии НИСАГ с генетической стресс-зависимой формой артериальной гипертензии по сравнению с нормотензивной линией крыс WAG. С применением метода спектроскопии ядерно-магнитного резонанса (ЯМР-спектроскопия) в образцах сыворотки крови было идентифицировано 56 метаболитов, при этом для 18 метаболитов выявлены достоверные различия по концентрации в сыворотке крови между линиями крыс. Статистический анализ полученных данных показал, что гипертензивный статус крыс НИСАГ характеризуется сочетанным повышением концентраций лейцина, изолейцина, валина, мио-инозитола, изобутирата, глутамата, глутамина, орнитина и креатинфосфата и понижением концентраций 2-гидроксиизобутирата, бетаина, тирозина и триптофана. Такие изменения концентраций метаболитов ассоциированы с характерными для гипертензивного статуса изменениями в регуляции метаболизма глюкозы (метаболомные маркеры – лейцин, изолейцин, валин и мио-инозитол), синтеза оксида азота (орнитин) и катехоламинов (тирозин) и с воспалительными процессами (метаболомные маркеры – бетаин, триптофан). Таким образом, идентификация метаболомного профиля стрессзависимой формы артериальной гипертонии представляется важным результатом, полезным для разработки персонализированного подхода к профилактике и лечению гипертонической болезни.

## Introduction

Hypertension is a complex multifactorial disease determined by both genetic and environmental factors, as well as the effects of genotype-environment interactions. Currently, a wide selection of antihypertensive drugs and their combinations is available for clinical medicine (Laurent, 2017). However, only a few of them are actually used (vasodilators, diuretics, blockers of certain receptors and ion channels): they affect the final links in the pathogenesis of arterial hypertension and usually do not address the initial etiological mechanisms of the disease. This can partly explain the fact that only 30 % of hypertensive patients successfully achieve and control blood pressure (BP) targets (Thoenes et al., 2010).

To improve the effectiveness of assigned therapy, objective criteria that enable positive identification of the individual etiological and pathogenetic characteristics of the disease are needed. First of all, of interest are genetic markers. Genes associated with arterial hypertension have been identified in numerous studies, including genome-wide analysis of a huge number of polymorphisms. However, these polymorphic loci account for only a small percentage (2–3 %) of BP variability in the tested populations (Hoffmann et al., 2017). Obviously, the contribution of environmental factors, as well as the effects of genotype-environment interaction, dominates. Non-additive intergenic interactions and epigenetic influences may also be of great importance (Toland et al., 2008; Niu et al., 2009; Friso et al., 2015).

In recent decades, along with the analysis of the genome and transcriptome, metabolomic and proteomic studies have been developed. Metabolic profiles of biological tissues represent the influence on the metabolism of both genes and the environment, which makes it possible to obtain an integral assessment of multifactorial influences. Therefore, the search for metabolic markers, along with genetic ones, provides a more comprehensive picture of pathogenetic processes occurring in a particular person, and also allows clustering patients according to various forms of hypertensive conditions. Awareness of the metabolic pathways underlying a particular type of arterial hypertension would make the treatment protocols more efficient (Byrd, 2016).

Comprehensive metabolomic studies of arterial hypertension pathogenesis are still few in number. However, hypertensive patients were found to have specific changes in the lipid profile of blood serum (Brindle et al., 2003), changes in carbohydrate metabolism – an increase in glucose and galactose levels and a decrease in fructose concentration (Liu et al., 2011), an increase in the concentration of alpha-1-acid glycoprotein, a marker of inflammatory processes (De Meyer et al., 2008). Some data were also obtained on the metabolic profile in the strain of rats with spontaneous hypertension – SHR: an age-related decrease in the concentrations of certain amino acids (serine, methionine, ornithine, phenylalanine) and an increase in the content of free fatty acids in blood plasma (Aa et al., 2010), reduced in comparison with normotensive control rats urinary citrate and alpha-ketoglutarate levels at 8 weeks of age (Akira et al., 2008), increased urinary taurine and creatine at 12 and 26 weeks of age (Akira et al., 2005).

In the present study, for the first time, we analyzed the metabolic profile of blood serum in experimental animals with hereditary stress-sensitive arterial hypertension – ISIAH rats.

## Materials and methods

Experimental animals. Male ISIAH rats with inherited stress-induced arterial hypertension (n = 10), control normotensive male WAG rats (Wistar Albino Glaxo) (n = 10), all aged 3–4 months. The experimental animals were kept under standard conditions in the conventional vivarium of the Institute of Cytology and Genetics (Siberian Branch of the Russian Academy of Sciences – SB RAS), receiving standard chow (Chara, Russia) and drinking water ad libitum. All procedures involving animals complied with the ethical standards approved by the legal acts of the Russian Federation, the principles of the Basel Declaration and the recommendations of the Inter-Institute Committee on Biological Ethics at the Institute of Cytology and Genetics (SB RAS) (protocol No. 127, September 8, 2022).

Blood pressure monitoring. Performed on a device for non-invasive blood pressure measurement (BIOPAC, USA) using the tail-cuff method, after preliminary adaptation of animals to this procedure for 3–4 days.

Blood serum sampling. Carried out during the euthanasia of experimental animals by decapitation. Collected peripheral blood was kept for an hour to form a primary clot, then centrifuged (+4 °C, 3000 rpm, 20 min), the obtained blood serum was stored at –70 °C.

Extraction of metabolites from blood serum samples. Performed at the Research Equipment Sharing Center “Mass-spectrometric Studies” of the International Tomography Center (SB RAS), at the Laboratory of Proteomics and Metabolomics. Metabolites were extracted using a mixture of methanol-chloroform-water in the ratio of 1:1:1, according to a previously developed protocol (Zelentsova et al., 2020; Fomenko et al., 2022). The volume of serum for the study was 300 μl. The lyophilized extracts were diluted in 600 μl of deuterated phosphate buffer (50 mM, pH 7.2) with the addition of internal standard DSS (2 × 10–5 M 3-(trimethylsilyl) propane-1-sulfonate sodium).

NMR spectra. Obtained on the AVANCE III HD 700 MHz NMR spectrometer (Bruker BioSpin, Germany) equipped with an Ascend cryomagnet with a field strength of 16.44 Tesla. The survey parameters are described in earlier articles (Zelentsova et al., 2020; Fomenko et al., 2022). MestReNova v12.0 software was used to process the spectra and integrate the signals.

Identification of metabolites in the studied samples. Carried out using the Human Metabolome Database (https:// hmdb.ca/) and our own data on the metabolic profiles of human and animal biological fluids (Tsentalovich et al., 2020; Fomenko et al., 2022).

Statistical processing of metabolomic data. Performed using the Statistica 8 software package (http://statsoft.ru/) and the MetaboAnalyst 5.0 web platform (https://www.metaboanalyst. ca/) (Pang et al., 2021), applying multivariate statistics (principal component analysis) and non-parametric method for assessing intergroup differences (Mann–Whitney U-test). Values at p < 0.05 were considered statistically significant.

## Results

As a result of the NMR spectra analysis, the concentrations of 56 metabolites were determined in the blood serum of ISIAH (BP = 205.6 ± 7.3 mm Hg) and WAG (BP = 136.6 ± 3.1 mm Hg) rats. Significant interstrain differences in serum concentrations of 18 metabolites were observed (see theTable).

**Table 1.**
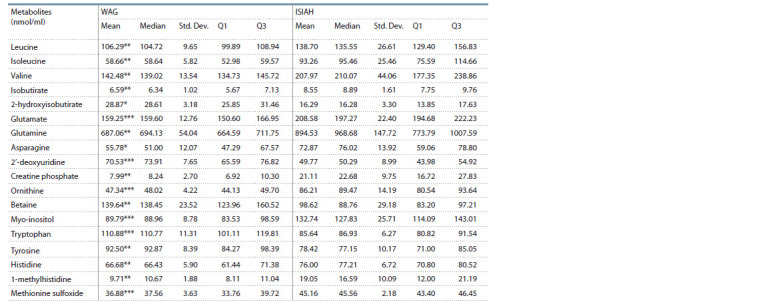
Serum metabolite concentrations in ISIAH and WAG rats * p < 0.05; ** p < 0.01; *** p < 0.001.

In ISIAH rats, the concentrations of leucine, isoleucine, valine, isobutyrate, glutamate, glutamine, asparagine, creatine phosphate, ornithine, myo-inositol, histidine, 1-methylhistidine, methionine sulfoxide in blood serum were significantly higher than in WAG rats, whereas the concentrations of 2-hydroxyisobutyrate, 2′-deoxyuridine, betaine, tryptophan, and tyrosine in ISIAH rats were decreased compared to normotensive controls.

In order to isolate metabolites that are associated with elevated blood pressure in ISIAH rats, a multivariate analysis was performed. Principal component analysis revealed two main factors (two axes) that together account for 47.2 % of the total variation in serum concentrations of the studied metabolites

**Fig. 1.**
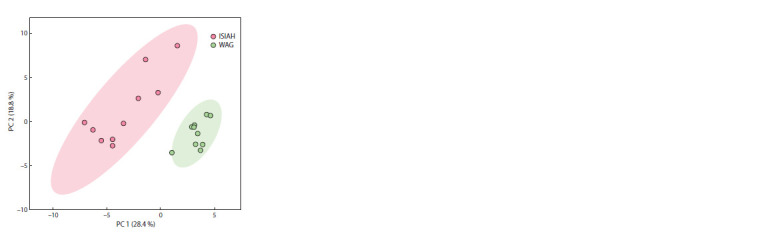
Location of hypertensive ISIAH rats and normotensive WAG rats in principal component coordinates (PC 1 and PC 2) obtained by analyzing the metabolic profile of blood serum using the MetaboAnalyst 5.0 web platform.

As can be seen from Fig. 1, the experimental animals were clustered in the space of two principal components on the basis of belonging to a hyper- or normotensive strain. The projections of these clusters on the axis of the first component practically do not overlap, while their projections on the axis of the second component coincide. Thus, the first principal component can be defined as the axis of presence/ absence of hypertensive status. In order to establish a relationship between the concentrations of the detected metabolites and the hypertensive status, it is necessary to consider their distribution against the first principal component. This is determined by the “loadings” that metabolites make on the first component.

**Fig. 2.**
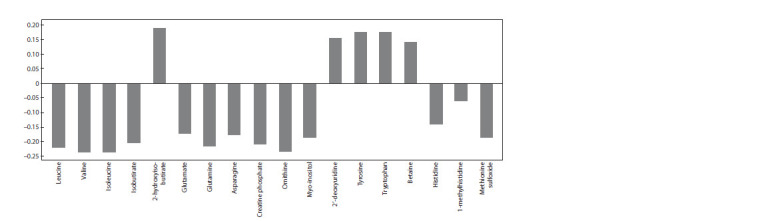
Loadings on the first principal component made by those metabolites, the concentrations of which in the blood serum significantly differed in ISIAH and WAG rats

Positive loadings on the axis of the first component were made by 2-hydroxyisobutyrate, tryptophan, tyrosine, betaine, 2′-deoxyuridine; ornithine, valine, isoleucine, leucine, isobutyrate, glutamate, glutamine, asparagine, creatine phosphate, myo-inositol, histidine, 1-methylhistidine, methionine sulfoxide made negative loadings (Fig. 2). Thus, the listed metabolites are largely responsible for the clustering of groups of experimental animals according to the level of their blood pressure.

## Discussion

**ВСАА, branched-chain amino acids**

Amino acids of the BCAA group – leucine, isoleucine and valine – are essential, and participate in the protein synthesis and degradation. They are also signal molecules in glucose metabolism, activating the mTORC1 complex, which phosphorylates the insulin receptor substrate IRS-1 (Yoshizawa, 2012; Yoon, 2016). Elevated plasma concentrations of BCAA amino acids have been associated with obesity, insulin resistance, decreased glucose tolerance, and type 2 diabetes, according to a number of studies (Newgard et al., 2009; Wang T.J. et al., 2011; Roberts et al., 2014). It has also been found that leucine, isoleucine, and valine are involved in the hypothalamic regulation of glucose metabolism in the liver (Arrieta-Cruz et al., 2016).

Prospective cohort studies involving a large number of patients (2243 (Hu et al., 2016) and 27,041 (Tobias et al.,2018)) show that elevated plasma concentrations of BCAA amino acids positively correlate with the risk of developing cardiovascular diseases (stroke, myocardial infarction, coronary disease). In ISIAH rats, a decrease in the level of immunoreactive insulin in the blood and glucose tolerance has been previously found, probably due to a genetically determined increased activity of the sympathoadrenal and thyroid systems (Shorin et al., 1990; Buzueva et al., 2006). Activation of the pancreas sympathetic innervation reduces insulin production by β-cells, acting through α2-adrenergic receptors (van Duk et al., 1995), and thyroid hormones affect insulin production through the regulation of insulin-like growth factor 1 secretion (Cavaliere et al., 1987). These data are consistent with the results of the present study: the concentrations of leucine, isoleucine, and valine in blood serum are significantly increased in ISIAH rats compared to controls (see the Table), which suggests that amino acids of the BCAA group can be considered as metabolic markers of hereditary stress-induced arterial hypertension

**Myo-inositol**

Some inositol isomers (particularly myo-inositol) have insulinlike properties and may reduce insulin resistance in patients with metabolic syndrome (Giordano et al., 2011; Croze, Soulage, 2013). It has been shown that myo-inositol plasma level is associated with BP level in patients with hypertension (Yang M. et al., 2016), and the use of myo-inositol as part of a dietary supplement for six months reduced the concentration of cardiovascular diseases biomarkers in menopausal women and in women with a history of metabolic syndrome (D’Anna et al., 2014). It is assumed that inositol derivatives affect the IP3 receptor, which regulates the contractility of the smooth muscle walls of blood vessels through L-type calcium channels (Abou-Saleh et al., 2013). An increased level of myo-inositol in the blood serum of ISIAH rats with hereditary stress-induced hypertension may indicate its involvement in the pathogenesis of the hypertensive status of rats of this strain.

**SCFA, short-chain fatty acids**

Short-chain fatty acids – formic, acetic, propionic, butyric, isobutyric, valeric, isovaleric and others – are produced in the large intestine during fiber fermentation, being an important source of energy for colonocytes, and having anti-inflammatory and antitumor properties (Andoh et al., 2003; Fernández et al., 2016). Short-chain fatty acids entering into acylation reactions can modify histones, thus regulating the expression of genes involved in the mechanisms of development of the metabolic syndrome, type 2 diabetes, and ischemic tissue damage (Sabari et al., 2017; Chen et al., 2020). Decreased production of short-chain fatty acids produced by gut bacteria leads to intestinal dysfunction, inflammation, kidney failure, and, as a result, to increased blood pressure (Kim et al., 2018; Felizardo et al., 2019). In SHR rats with spontaneous hypertension, elevated BP has been associated with a reduced content of acetate and butyrate-producing bacteria in the intestinal microbiota (Yang T. et al., 2015).

The relationship between BP levels and various acids of the SCFA group in salt-sensitive Dahl rats has also been studied: a high salt load resulted in an increase in the concentration of acetate, propionate and isobutyrate in fecal samples (Bier et al., 2018). Mechanisms of this relationship have not yet been studied in detail, but there is evidence that short-chain fatty acids can affect vessels and kidneys through endothelial receptors associated with G-proteins, which leads to a change in BP levels (Natarajan et al., 2016). In hypertensive ISIAH rats, a change in the ratio of SCFAs and their derivatives was also observed when compared with the normotensive control: isobutyrate blood concentration was significantly increased, while 2-hydroxyisobutyrate levels were decreased (see the Table).

**Glutamate, glutamine**

Associations of glutamate and glutamine concentrations, as well as hepatic aspartate aminotransferase activity, with insulin resistance and the development of the metabolic syndrome have been shown (Sookoian, Pirola, 2012). There is also evidence that plasma glutamate is positively correlated with blood pressure, body mass index, insulin and triglyceride levels. The glutamine/glutamate ratio is inversely related to these parameters (Liu X. et al., 2019). Considering that ISIAH rats in this study have increased serum levels of both glutamate and glutamine when compared to control WAG rats, but the glutamine concentration (894.53 nmol/ml) is several times higher than the glutamate content (208.58 nmol/ml), interpretation of observed interstrain differences in glutamate and glutamine concentrations requires more research.

Glutamate and glutamine also contribute to the metabolism of arginine and ornithine, which are involved in the urea and nitric oxide cycle (Wilson et al., 2001). Ornithine concentration in the serum of ISIAH rats is also increased compared to the control. It is established that α-difluoromethylornithine administration resulted in the restoration of endothelial function and prevented an increase in blood pressure in spontaneously hypertensive SHR rats (Demougeot et al., 2005). In an earlier SHR study, α-difluoromethylornithine reduced the rate of aortic and caudal artery contraction in response to electrical stimulation and norepinephrine administration, while a decrease in arterial wall thickness and a decrease in the content of polyamines in vessels was also observed (Soltis et al., 1994).

**Metabolites associated with inflammation**

In a study involving healthy volunteers (323 people) and ischemic stroke patients (323 people), choline, like its metabolite betaine, was found to reduce the risk of cardiovascular complications (Zhong et al., 2021). Long-term use of choline and betaine as a dietary supplement was also shown to lower blood pressure in hypertensive patients (Golzarand et al., 2021). Intragastric administration of betaine to rats modeling pulmonary hypertension resulted in a decrease in right ventricular and pulmonary artery blood pressure, in a decrease in the degree of ventricular hypertrophy and in remodeling of the arterial wall, presumably due to anti-inflammatory action – betaine also reduced the levels of MCP-1, ET-1, NF-κB, TNF-α, IL-1β (Yang J.M. et al., 2018).

Tryptophan is an essential aromatic amino acid. In mammals, tryptophan is metabolized in three partially overlapping pathways. The main pathway – kynurenine pathway – includes oxidation and destruction of the indole ring, producing derivatives: kynurenic and anthranilic acids. One of the 60 tryptophan molecules is converted into nicotinic acid (vitamin B3, niacin). The second pathway is the serotonin pathway, where tryptophan is converted to serotonin and melatonin. The third pathway is the indole pathway, the formation of indole derivatives, which are then excreted in the urine (Richard et al., 2009). It has been shown that disorders in the links of the kynurenine pathway facilitate development of cardiovascular diseases, including an increase in blood pressure (Song et al., 2017; Verheyen et al., 2017). It is possible that tryptophan and kynurenine promote vasodilation through participation in the adenylate cyclase and guanylate cyclase systems of secondary intracellular messengers, triggering a cascade of reactions leading to the activation of nitric oxide receptors and to a decrease in the concentration of Ca2+ ions in the smooth muscle walls of blood vessels (Lincoln et al., 1990; Stasch et al., 2006; Wang Y. et al., 2010).

Betaine and tryptophan concentrations were significantly reduced in the blood serum of hypertensive ISIAH rats compa- red with normotensive control, which may indicate that inflammatory processes play a role in establishing and maintaining the hypertensive status of ISIAH rats. Recently, there has been even more evidence of the important role of vascular wall inflammation in the pathogenesis of hypertensive conditions, including those involving interleukins IL-1β and IL-18 (Patrick et al., 2021).

**Metabolites associated with energy processes**

Creatine phosphate is a source of rapidly mobilized energy in tissues where energy metabolism is most intense – skeletal muscles, myocardium, brain. Due to the fact that direct transport of ATP across the mitochondrial membrane is difficult, creatine phosphate serves as a “shuttle”, participating in the transport of chemical energy between mitochondria and energy-consuming areas. ATP with mitochondrial creatine kinase phosphorylates creatine to creatine phosphate, which goes, for example, to myofibrils. Myofibrillic creatine kinase forces creatine phosphate to phosphorylate ADP to ATP, producing creatine, which again returns to the mitochondria, and the cycle repeats (Bessman, Carpeneter, 1985).

Changes in the content and ratio of creatine and phosphocreatine in tissues can be a signal of various pathologies (Strumia et al., 2012). It has been shown that a decrease in the ratio of creatine phosphate/ATP correlates with the severity of heart failure (Neubauer et al., 1992) and with the severity of myocardial hypertrophy (Ye et al., 2001). It is also known that exogenous creatine phosphate administration has a cardioprotective effect on the ischemic myocardium (Scattolin et al., 1993; Azova et al., 2015; Zhang et al., 2015). In our study, in ISIAH rats, serum creatine phosphate concentration was increased nearly three-fold compared with the normotensive control. To explain this difference in peripheral concentrations of creatine phosphate, additional studies are required, including an assessment of creatine phosphate concentration and the ratio of creatine phosphate/ATP in the myocardium of hypertensive ISIAH rats.

**Metabolites associated with the synthesis of catecholamines**

Tyrosine is an aromatic amino acid from which, via enzyme tyrosine hydroxylase, catecholamines are synthesized: dopamine, adrenaline, norepinephrine. Catecholamines are the main effectors of the sympathoadrenal system, affecting cardiac output and vascular resistance (Lee et al., 2016). The main indicators of the sympathoadrenal system functions are catecholamine concentrations and tyrosine hydroxylase activity (Yamabe et al., 1973; Moura et al., 2005), but tyrosine concentration may also be considered as a marker of catecholamine synthesis disorders: for example, in a metabolomic study of urine samples from patients with hypertensive nephrosclerosis, a decrease in tyrosine and dopamine levels has been found (Ovrehus et al., 2019).

It has previously been shown that the production of epinephrine by the adrenal glands and norepinephrine in the brain is increased in ISIAH rats compared with WAG (Markel et al., 2007; Redina et al., 2021), which allows to suggest that the reduced serum tyrosine level in ISIAH rats is a marker of changes in catecholamine synthesis.

## Conclusion

Thus, we conclude that the metabolic profile of blood serum, which indicates the presence of a stress-dependent form of arterial hypertension, can be described as follows: an increase in the concentrations of leucine, isoleucine, valine, myo-inositol, isobutyrate, glutamate, glutamine, ornithine, creatine phosphate, and a decrease in the concentrations of 2-hydroxyisobutyrate, betaine, tryptophan, tyrosine. Elevated concentrations of leucine, isoleucine, valine, and myo-inositol are associated with glucose metabolism and insulin resistance observed in ISIAH rats (Shorin et al., 1990; Pivovarova et al., 2020). Ornithine plays an important role in the urea synthesis, and is also associated with the metabolism of arginine and the production of vasoactive factor – nitric oxide; therefore, its consideration as a metabolic marker of hypertension pathogenesis seems to be quite reasonable. Betaine is described as having an anti-inflammatory effect in various pathologies (Zhao et al., 2018), therefore, a decrease in its concentration in the serum of ISIAH rats may indicate the involvement of the inflammatory process in the pathogenesis of arterial hypertension. Serum tryptophan may play the same role as a negative marker of the inflammatory process (Sorgdrager et al., 2019); its decrease in ISIAH rats may have a pro-inflammatory effect.

The results obtained are the starting point for a more detailed study on the association of these metabolic markers with the development of hypertensive status at certain stages of the stress-dependent arterial hypertension pathogenesis.

## Conflict of interest

The authors declare no conflict of interest.
